# Bioelectrical impedance vector analysis (BIVA) for measuring the hydration status in young elite synchronized swimmers

**DOI:** 10.1371/journal.pone.0178819

**Published:** 2017-06-07

**Authors:** Marta Carrasco-Marginet, Jorge Castizo-Olier, Lara Rodríguez-Zamora, Xavier Iglesias, Ferran A. Rodríguez, Diego Chaverri, Daniel Brotons, Alfredo Irurtia

**Affiliations:** 1 INEFC-Barcelona Sports Sciences Research Group, National Institute of Physical Education of Catalonia, Universitat de Barcelona, Barcelona, Spain; 2 Department of Health Sciences, Mid Sweden University, Östersund, Sweden; 3 Swedish Winter Sports Research Centre, Mid Sweden University, Östersund, Sweden; 4 Sport and Health Area of the Catalan Sport Council, Government of Catalonia, Barcelona, Spain; Nanyang Technological University, SINGAPORE

## Abstract

**Purpose:**

The assessment of body hydration is a complex process, and no measurement is valid for all situations. Bioelectrical impedance vector analysis (BIVA) has emerged as a relatively novel technique for assessing hydration status in sports. We applied BIVA a) to determine hydration changes evoked by an intense synchronized swimming (SS) training session; b) to characterize the sample of young elite swimmers in relation with a nonathletic reference population; and c) to generate its 50%, 75% and 95% percentiles of the bioelectrical variables.

**Methods:**

Forty-nine elite SS female swimmers of two age categories, comen (C_o_: 13.9 ± 0.9 years, n = 34) and junior (J_r_: 16.3 ± 0.6 years, n = 15), performed a long, high intensity training session. Body mass (BM) and bioelectrical variables (R, resistance; Xc, reactance; PA, phase angle; and Z, impedance module) were assessed pre- and post-training. BIVA was used to characterize 1) the distribution pattern of the bioelectrical vector (BIA vector) for both age groups, and 2) pre- to post-training BIA vector migration. Bioelectrical variables were also correlated with BM change values.

**Results:**

Most swimmers were mostly located outside the 75% and some beyond the 95% percentile of the bioelectrical tolerance ellipses of the general population. The BIA vector showed statistically significant differences in both C_o_ (*T*^*2*^ = 134.7, *p* = 0.0001) and J_r_ (*T*^*2*^ = 126.2, *p* < 0.001). Both groups were also bioelectrically different (*T*^*2*^ = 17.6, *p* < 0.001). After the training session, a decrease in BM (*p* = 0.0001) and an increase in BIA variables (*p* = 0.01) was observed. BIVA also showed a significant pre-post vector migration both in C_o_ (*T*^2^ = 82.1; *p* < 0.001) and J_r_ (*T*^2^ = 41.8; *p* < 0.001). No correlations were observed between BM changes and bioelectrical variables.

**Conclusions:**

BIVA showed specific bioelectrical characteristics in young elite SS athletes. Considering the decrease in BM and the migration of the BIA vector, we conclude that the homeostatic hydration status of these young elite female swimmers was affected by the execution of intense training sessions. From a methodological perspective, BIVA appears to be sensitive enough to detect subtle hydration changes, but further research is needed to ensure its validity and reliability. Moreover, these findings highlight the importance of ensuring adequate fluid intake during training in young SS athletes.

## Introduction

Since becoming part of the Olympic program in 1984, synchronized swimming has enjoyed a growing worldwide popularity. This highly technical sport combines aerobic and anaerobic endurance, flexibility, strength, power, acrobatics and performance skills, and choreography [1] requiring long hours of training to attain such broad attributes [2].

Most synchronized swimmers enter the sport as young girls at the recreational level, and by the age of 13–15 years, the more talented athletes start training and competing at a more intense level [3]. Elite swimmers tend to train 6 days per week with one day off, and training sessions usually last between 3 and 5 hours [2, 3] and are divided in two workouts per day with different content. For example, sport-specific skill training in the water could follow a pool session of swimming for aerobic fitness. A dry land training could occur later in the same day, consisting of flexibility, dry land drills, or a psychology session [3]. As a result, training demands at the elite level often result in high-volume—averaging approximately 40 h per week—and high-intensity training programs [2, 4].

Young athletes may experience fluid imbalances if some conditions are met, with possible consequences on their physical performance, cognitive performance and health maintenance [5–8].

Despite the high requirements at such a young age, information about fluid intake and hydration during the strenuous SS training is scarce. Female swimmers show low energy availability, especially in phases of intensified training performed before competition [9]. Findings highlight the importance of ensuring adequate fluid intake during synchronized swimming training to enable optimal performance. Nevertheless, it has been suggested that there is lower fluid replacement during pool sessions, possibly due to the limited drink breaks or because athletes try to avoid potential gastrointestinal discomfort if the exercise requires them to be upside down [10].

The assessment of body hydration is a dynamic and complex process, and no measurement is valid for all situations [11]. In this context, bioelectrical impedance vector analysis (BIVA) emerges as a relatively novel technique for assessing hydration status without algorithm-inherent errors or requiring assumptions such as constant tissue hydration [12, 13]. BIVA uses raw bioelectrical impedance parameters, i.e., resistance (R, the opposition to flow through intra- and extracellular ionic solutions) and reactance (Xc, additional opposition from the capacitance effect of cell membranes and tissue interfaces), standardized by height (h) to remove the effect of conductor length, which yields a vector that is plotted in an RXc graph [14]. Overall, BIVA properties are especially interesting for hydration assessment in sports, during both competitions and training [15, 16].

The aim of this study was, first, to determine the hydration changes evoked during a synchronized swimming training session by focusing on changes of the whole-body impedance vector. Secondly, we compared the SS young elite sample with a reference nonathletic population and generated its 50%, 75% and 95% percentiles of the bioelectrical variables distribution, also known as tolerance ellipses. We hypothesized that the hydration status of the young swimmers would be altered by the long, intense training sessions and the barriers for an adequate fluid intake. In this line, these swimmers would be characterized by a specific distribution of BIVA variables when compared to the reference population.

## Materials and methods

### Participants

Eighty-four female SS athletes of two competitive categories, comen (C_o_, n = 53) and junior (J_r_, *n* = 31) swimmers, including the entire Spanish national junior team, were recruited for the study in March 2012. Thirty-five (C_o_, n = 19; J_r_, *n* = 16) did not meet inclusion criteria. Inclusion criteria were as follows: (1) to have competed at national and/or international level at least in the previous two years; (2) to not present injuries or any clinical condition at the time of the study; (3) to be in a postmenarcheal state with the ovarian cycle between days 5^th^ to 11^th^ [17]; (4) to not be under contraceptives or menstrual cycle pharmacological regulators treatment. Sample size was calculated to detect an effect size (ES) = 0.5, with an estimated sample standard deviation (SD) = 7.0, and a SD for changes = 0.7, requiring a minimum of 15 subjects per group. Power (P = 1 –β) was set at 0.80, and the confidence interval was α = 0.05. Forty-nine athletes were selected (C_o_, n = 34; J_r_, n = 15). All subjects voluntarily participated in the study and delivered written informed consent, with parental permission when needed. The study was conducted following the WMA Helsinki Declaration Statement [18] and approved by the Ethics Committee for Clinical Sport Research of Catalonia. The characteristics of the participants are shown in Table 1.

**Table 1:** Characteristics of participants.

		All swimmers _(95% CI)_	Comen _(95% CI)_	Junior _(95% CI)_	Unpaired t-test	
		*n* = 49	*n* = 34	*n* = 15	*t*	*p*
General						
	Age (years)	14.6 ± 1.4 _(14.2–15.0)_	13.9 ± 0.9 _(13.6–14.2)_	16.3 ± 0.6 _(16.0–16.7)_	-10.851	0.0001^*^
	Training (h/week)	19.4 ± 7.6 _(17.4–21.8)_	15.0 ± 2.7 _(14.0–15.9)_	30.0 ± 3.8 _(28.0–32.1)_	-15.911	0.0001^*^
	Practice (years)	6.9 ± 1.8 _(6.4–7.4)_	5.9 ± 1.1 _(5.6–6.3)_	9.1 ± 1.0 _(8.6–9.7)_	-9.980	0.0001^*^
Anthropometric						
	Height (cm)	163.3 ± 7.6 _(161.1–165.4)_	161.9 ± 8.2 _(159.0–164.8)_	166.3 ± 4.8 _(163.7–169.0)_	-1.943	0.058
	BM (kg)	49.1 ± 7.0 _(47.1–51.2)_	47.2 ± 7.0 _(44.8–49.7)_	53.5 ± 5.2 _(50.6–56.3)_	-3.103	0.003^*^
	BMI (kg/m^2^)	18.4 ± 1.8 _(17.9–18.9)_	18.0 ± 1.9 _(17.3–18.6)_	19.3 ± 1.3 _(18.6–20.0)_	-2.514	0.015^*^
	Fat mass (%)	16.5 ± 4.4 _(15.2–17.8)_	15.6 ± 4.7 _(13.9–17.2)_	18.6 ± 2.6 _(17.2–20.1)_	-2.382	0.021^*^
	Muscle mass (%)	38.0 ± 4.7 _(36.7–39.4)_	37.7 ± 5.4 _(35.8–39.6)_	38.8 ± 2.6 _(37.3–40.2)_	-0.722	0.474
Bioelectrical						
	R/h (Ω/m)	319.7 ± 36.7 _(309.1–330.2)_	328.4 ± 38.8 _(314.9–341.9)_	299.9 ± 21.6 _(287.9–311.9)_	3.286	0.002^*^
	Xc/h (Ω/m)	39.9 ± 3.9 _(38.7–41.0)_	40.0 ± 4.5 _(38.4–41.5)_	39.6 ± 2.2 _(38.4–40.8)_	0.395	0.695
	PA (°)	7.1 ± 0.5 _(7.0–7.3)_	7.0 ± 0.5 _(6.8–7.1)_	7.5 ± 0.4 _(7.3–7.7)_	-4.166	0.0001^*^

Values are mean ± SD; BM, body mass; BMI, body mass index; R, resistance; Xc, reactance; PA, phase angle; h, height; CI, 95% confidence interval

* significant differences between comen and junior swimmers (p < 0.05).

### Study design

This pre-post quasi-experimental study was both descriptive and correlational and aimed to approach the topic from an ecological perspective. The study analyzed the acute adaptations induced by synchronized swimming training session on body mass–BM (kg), bioelectrical vector variables [resistance (R, Ω), resistance adjusted by height (R/h, Ω/m), reactance (Xc, Ω), reactance adjusted by height (Xc/h, Ω/m), impedance module (Z, Ω), and phase angle (PA, °)] and the extracellular water/total body water ratio (ECW:TBW, %). In addition to these independent variables, several others were recorded to characterize the sample (Table 1) and the training (Table 2).

**Table 2:** Characteristics of the training sessions.

	All swimmers _(95% CI)_	Comen _(95% CI)_	Junior _(95% CI)_	Unpaired t-test	
	*n* = 49	*n* = 34	*n* = 15	*t*	*p*
Duration (min)	167.6 ± 28.0 _(159.6–175.7)_	149.6 ± 3.3 _(148.5–150.8)_	208.4 ± 10.3 _(202.7–214.1)_	-21.695	0.001^*^
Internal training load					
RPE (a.u)	6.6 ± 0.5 _(6.4–6.7)_	6.4 ± 0.5 _(6.3–6.6)_	6.8 ± 0.6 _(6.5–7.1)_	-2.220	0.03^*^
Session–RPE	1102.4 ± 231.3 _(1036.0–1168.9)_	963.9 ± 78.5 _(963.5–991.3)_	1416 ± 129 _(1344.8–1488.0)_	-12.572	0.001^*^
Water intake (L)	0.6 ± 0.2 _(0.5–0.6)_	0.5 ± 0.2 _(0.4–0.6)_	0.7 ± 0.3 _(0.5–0.8)_	-2.177	0.04^*^

Values are mean ± SD; RPE, rating of perceived exertion (CR-10 scale); a.u, arbitrary units; CI, 95% confidence interval

* significant differences between comen and junior swimmers (p < 0.05).

### Procedures

**Fig 1:**
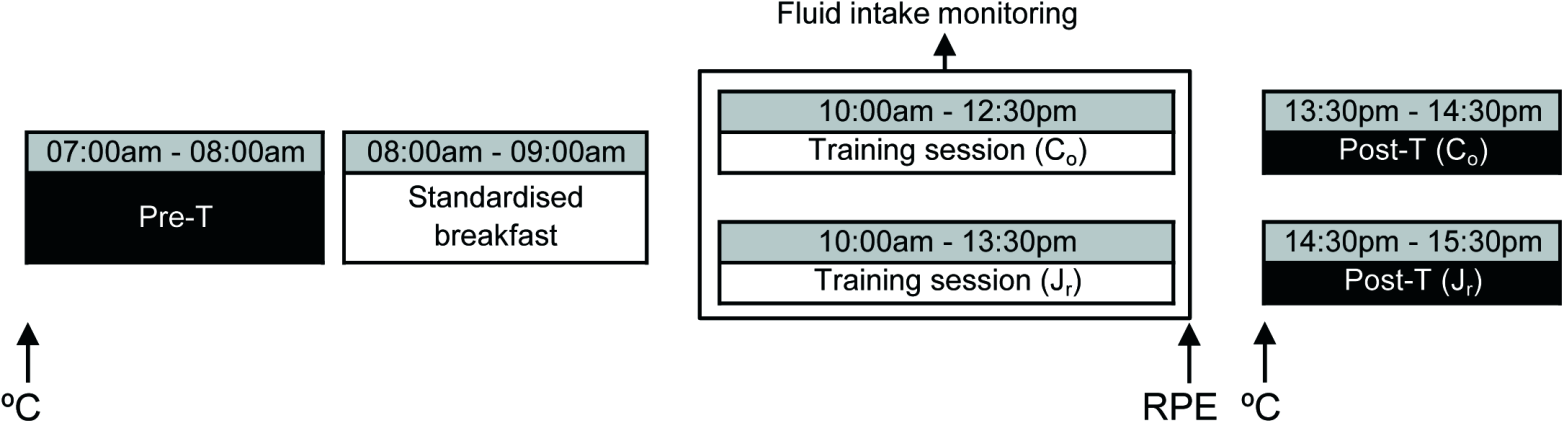
Study protocol. °C, body and skin temperature measurements; Pre-T, pre-training measurements; Post-T, post-training measurements; RPE, rating of perceived exertion; C_o_, comen; J_r_, junior.

The study was conducted two weeks before the Spanish National Synchronized Swimming Championship, within the 4-week precompetitive mesocycle. One training session was performed by each group on the same day. The protocol is chronologically summarized in Fig 1.

To attain a state of euhydration prior to BIA measurements [6], swimmers were required to abstain from caffeine, alcohol and exercise the day before the investigation [19]. They were also instructed to drink 3.0 L of fluid over 24 h (2.0 L to be consumed between 6:00 p.m. and 10:00 p.m.) in addition to their habitual western dietary practices. From 10:00 p.m. until the start of the pre-test next morning, no further fluid or food intake was allowed [6]. From 7:00 a.m. to 8:00 a.m. the following day, after monitoring body and skin temperatures, pre-training measurements were performed in a thermoneutral room (25°C) to obtain anthropometric (BM) and bioelectrical data (R, R/h, Xc, Xc/h, PA, Z, and ECW:TBW). Immediately after, participants consumed a standardized breakfast consisting of 1 cheese and ham sandwich, 1 plain yogurt, 1 banana, and 220 mL of natural orange juice [20]. At 10:00 a.m., all swimmers performed a category-specific training session in a 50-m indoor pool with 30 m available for use (water temperature: 25–26°C). The characteristics of both training are shown in Table 2.

Fluid intake (H2O) during the training was monitored by a certified dietician. Swimmers were instructed to drink a similar amount of water in the middle and at the end of training. Pre- and post-training, BIA measurements were conducted after urination and defecation [21] to minimize the influence of food/fluid ingestion [22] and exercise [23]; pre-training measurements were conducted while fasting, and the post-training data were obtained within the first hour of recovery. Just after completing the training session, the rating of perceived exertion (RPE) was assessed using the Borg CR-10 scale [24]. Finally, after checking that body and skin temperatures were similar to those registered in the pre-training measurements, the post-training assessment was performed.

#### Anthropometric assessment

BM was measured to the nearest 0.05 kg using a calibrated weighing scale (Seca 710, Hamburg, Germany). Height (h) was measured to the nearest 1 mm using a telescopic stadiometer (Seca 220, Hamburg, Germany). Body mass index (BMI) was calculated as body mass / height2 (kg/m^2^). Anthropometric measurements were taken according to the standard criteria of The International Society for the Advancement of Kinanthropometry [25].

#### Whole-body bioimpedance assessment

R and Xc were measured using a previously calibrated plethysmograph (Z-Metrix, BioparHom, Le Bourget-du-Lac, France) that emitted a 77 μA alternating sinusoidal current at seven operating frequencies (1, 5, 50, 150, 200, 250, and 325 kHz). The 50-kHz single frequency was selected for BIVA [26]; meanwhile, multi-frequency capabilities were used to estimate body composition–fat mass (FM) and muscle mass (MM), and the ECW:TBW was calculated by ECW/TBW•100. The device provides impedance values with an accuracy average error of 0.95 ± 1.58% and average repeatability errors of 0.55 ± 0.38% for all the frequency range (1 to 1000 kHz) [27]. Bioelectrical measurements were conducted under controlled conditions [14] through the standard whole-body, tetrapolar, distal BIA technique [28]. The anatomical sites for electrodes (Red Dot 2660–5, 3M Corporate Headquarters, St. Paul, MN, USA) were marked with a waterproof pen [29]. Bioelectrical measurements were repeated until they were stable to within 1 Ω (usually up to three times within an interval of 20–30 s). The average value was used in calculations [21].

Regarding the BIVA method, the correlation between R and Xc determines the ellipsoidal form of the bivariate probability distributions (confidence intervals for average vectors and tolerance for individual vectors). The vector direction is defined as the phase angle (PA) and is the geometric relationship between R and Xc. PA has been validated as an indicator of cellular health [12, 28] and has been interpreted as an index of fluid distribution between the intracellular and extracellular compartments [30], showing an inverse correlation with the ECW:TBW [31]. On the other hand, the length of the vector indicates hydration status from fluid overload (decreased resistance, short vector) to exsiccosis (increased resistance, longer vector), and a sideways migration of the vector due to low or high reactance indicates a decrease or increase in the dielectric mass (membranes and tissue interfaces) of soft tissues [32]. The individual vector can be ranked on the RXc point graph with regard to tolerance ellipses representing 50%, 75% and 95% according to the values of a reference population [14]. A comparison between the mean vectors of different samples with the 95% confidence ellipses can be performed on the RXc mean graph. Furthermore, the mean vector displacement of a group with the 95% confidence ellipse pre- to post-intervention was plotted on the RXc paired graph [33].

#### Temperature assessment

Core (°C_core_) and skin temperatures of the right hand (°C_hand_) and foot (°C_foot_) were measured using thermistors connected to a data logger (Squirrel 2010, Grant Instruments Ltd, Cambridge, UK). All swimmers were instructed to take a cold shower (as cold as tolerable) for 10–15 minutes post-training, in order to reduce cutaneous blood flow and temperature and remove accumulated electrolytes [34]. Skin temperature, as a surrogate for cutaneous blood flow [35], was measured just before BIA measurements; this verified the return to temperatures close to the pre-training values (*p* < 0.05): Pre-°C_core_: 36.8 ± 0.2°C vs. Post-°C_core_: 37.2 ± 0.3°C; Pre-°C_hand_: 29.6 ± 0.8°C vs. Post-°C_hand_: 29.2 ± 1.1°C; Pre-°C_foot_: 29.0 ± 1.2°C vs. Post-°C_foot_: 28.6 ± 1.0°C. Ambient air temperature and relative humidity in the indoor pool area were also controlled (27.5 ± 0.5°C and 64.5 ± 1.5%, respectively).

#### Internal training load assessment

The individual session-RPE (s-RPE) was chosen for rating the perceived exertion during training [36]. The CR-10 RPE scale [24] was shown to the swimmers immediately after the training was completed. Scores were computed by multiplying the duration of the training by the relative RPE values. One week before the study, all participants were assessed repeatedly during at least 3 training to disclose learning effects and to improve the consistency of the measurements [37].

### Statistical analysis

Descriptive statistics (mean, SD) were calculated for each independent variable and age category. Once the data were tested for normality (Shapiro-Wilks test), differences in anthropometric (BM) and bioelectrical variables (R, Xc, R/h, Xc/h, PA and Z) between pre- and post-training were analyzed by the Student’s paired *t*-test. The Student’s unpaired *t*-test was used to analyze group differences between age categories. Whole-body bioimpedance vectors were analyzed by the RXc graph method [14] using the BIVA software [38]. Each swimmer was plotted in the tolerance ellipses (50%, 75% and 95%) of the 14- to 15-year-old healthy female Italian reference population [39] as this was the reference population closest in age to our sample. The BIVA mean graph was performed to compare whole-body vectors of C_o_ vs. J_r_ groups, and each SS group vs. the reference population. The BIVA paired graph was used to analyze pre- to post-training changes in the vectors of C_o_ and J_r_. To examine the magnitude of pre-post ratio changes in anthropometric and bioelectrical variables, delta values (Δ, % of pre) were calculated. To estimate the relevance of these changes, relative ES were calculated using Cohen’s d. According to Cohen [40], ES was defined as small, d ≤ 0.2; medium, d ≤ 0.5; and large, d ≤ 0.8. Pearson’s correlation coefficient was used to determine possible statistical associations between a) PA vs. chronological age and PA vs. the ECW:TBW; and b) ΔBM vs. BIA vector variables (ΔR/h, ΔXc/h, ΔPA, ΔZ). A paired one-sample Hotelling’s *T*^2^ test was used to analyze pre- to post-training changes in the vector through the 95% confidence ellipses. A two-sample Hotelling’s *T*^2^ test was used to determine the BIA vector differences between C_o_ and J_r_ and between each SS group vs. the reference population. *P* < 0.05 was considered significant.

## Results

### Determinants of BIA vector distribution pattern in synchronized swimmers

**Fig 2:**
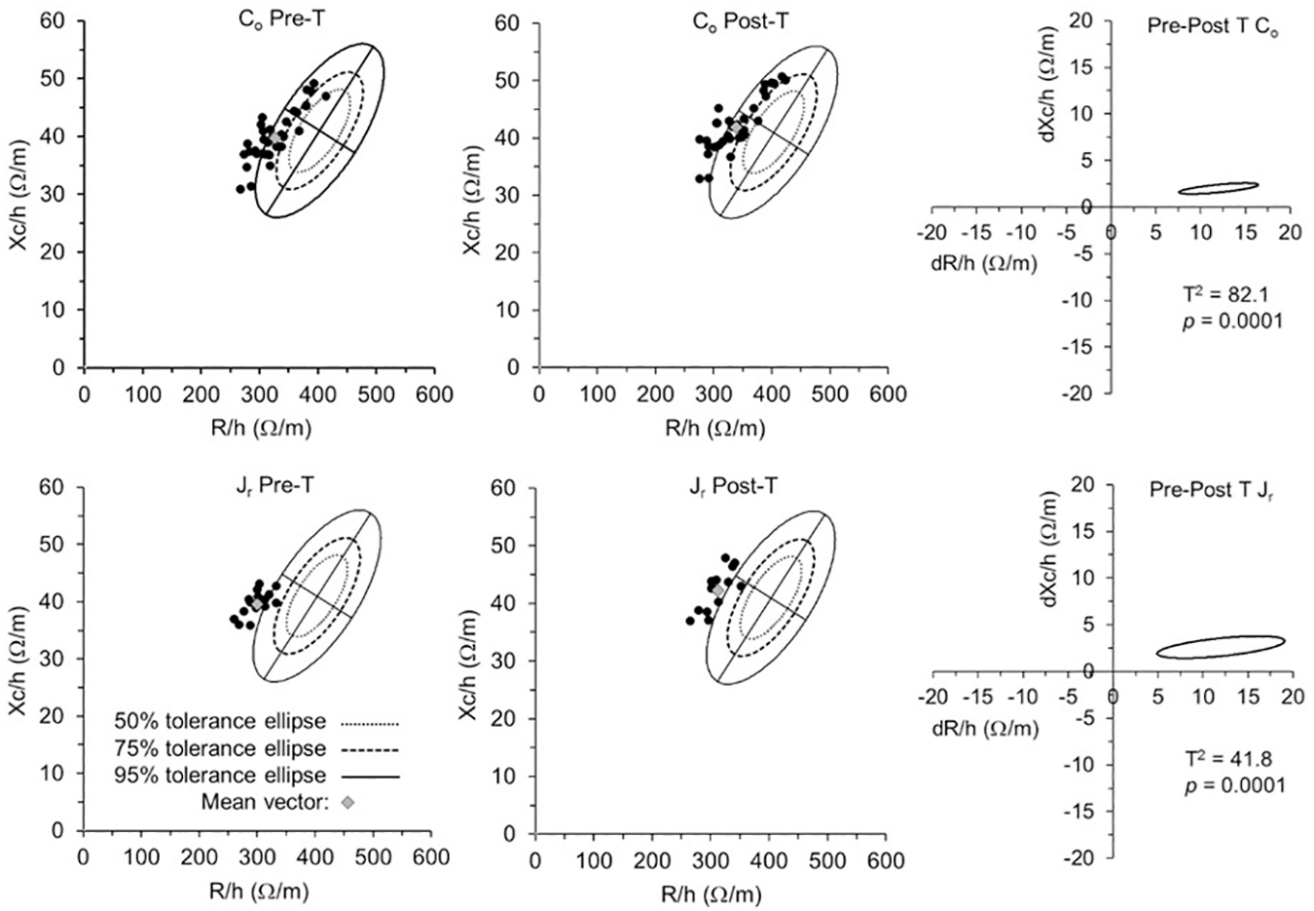
BIVA patterns before and after training. On the left side, scattergrams of the C_o_ and J_r_ individual (as well as the mean) impedance vectors, plotted on the 50%, 75%, and 95% tolerance ellipses of the corresponding healthy female reference population [39] are displayed both for pre- and post-training (Pre-T and Post-T, respectively). On the right side, mean vector displacements of C_o_ and J_r_ from pre- to post-training are shown. R/h, height-adjusted resistance; Xc/h, height-adjusted reactance; *T*^2^, Hotelling’s *T*^2^ test; *p*-value (significance at *p* < 0.05).

**Fig 3:**
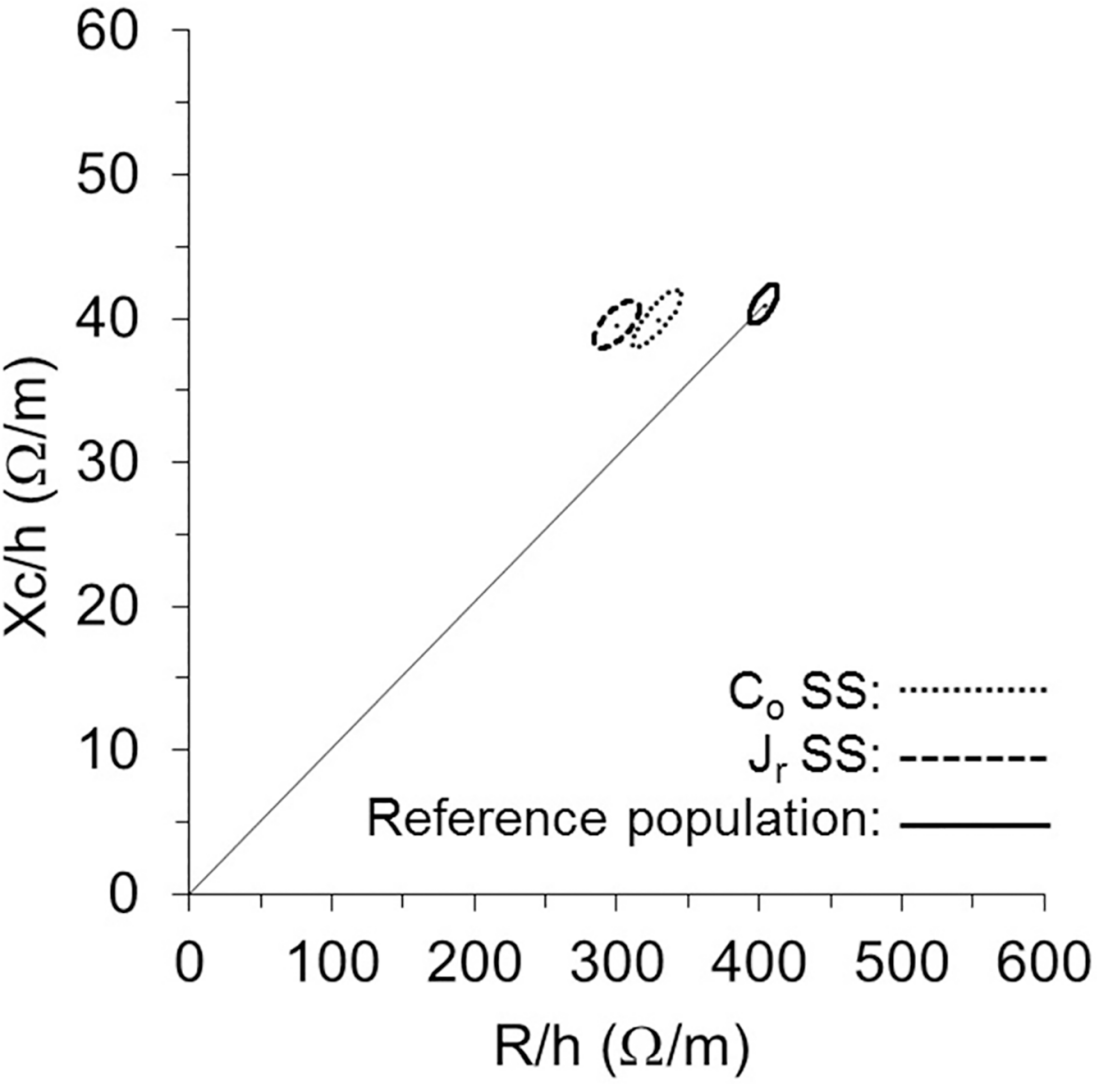
RXc mean graph. The 95% confidence ellipses for the mean impedance vectors of C_o_ (dotted line ellipse), J_r_ (dark dashed line ellipse) and the healthy female reference population (solid line ellipse with vector) [39] are shown. R/h, height-adjusted resistance; Xc/h, height-adjusted reactance; C_o_, comen; J_r_, junior; SS, synchronized swimmers.

The BIVA point graph (Fig 2) indicated that swimmers fell mostly outside the 75% tolerance ellipse regardless of age or competition level; in many cases, they were outside the 95% tolerance ellipse, denoting a higher density of body cell mass (BCM) than the reference population. Differences in the BIA vector in comparison with the reference population were found for C_o_ (*T*^2^ = 134.7, *p* = 0.0001) and J_r_ (*T*^2^ = 126.2, *p* < 0.001), as well as between both groups of SS swimmers (*T*^2^ = 17.6, *p* < 0.001) (Fig 3).

**Fig 4:**
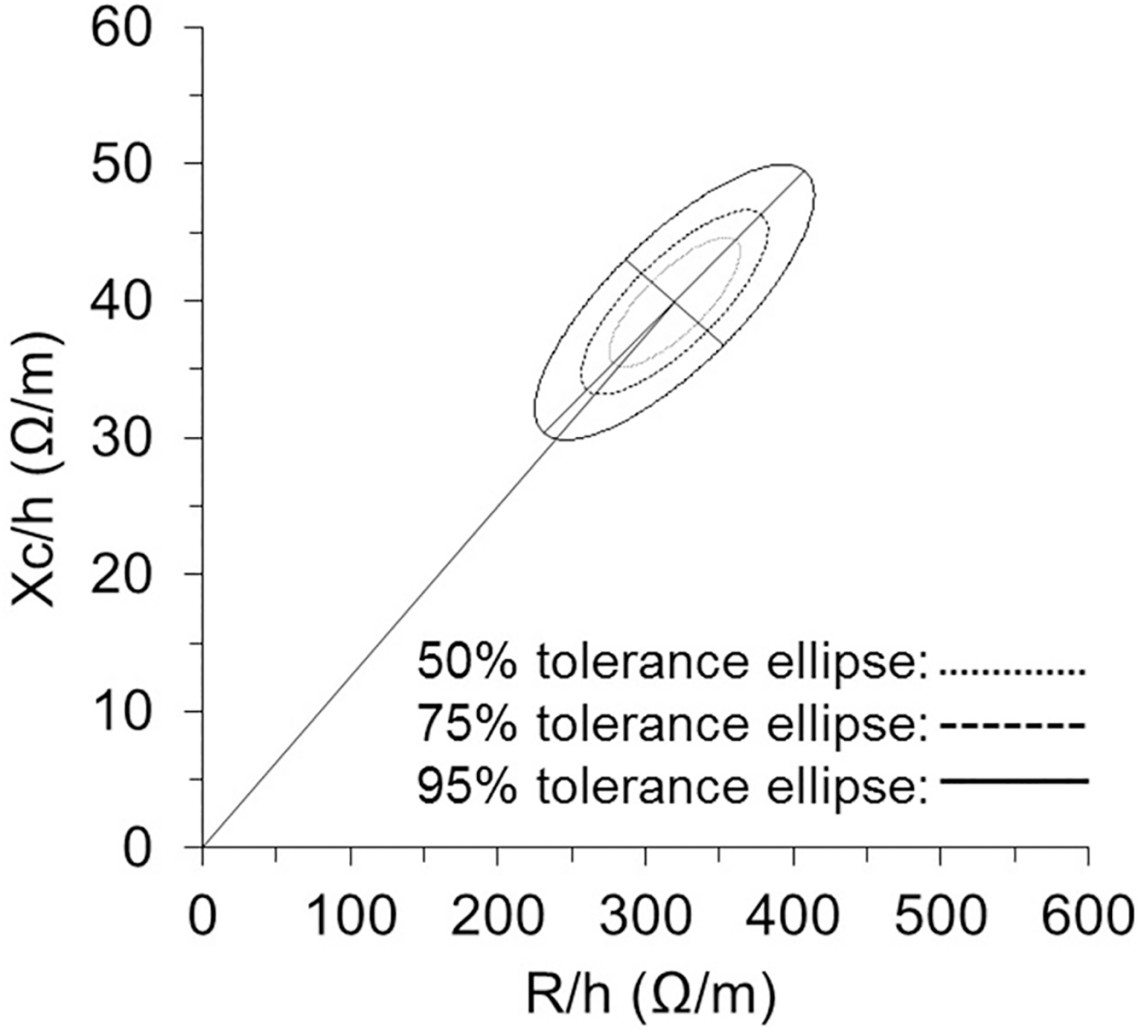
Tolerance ellipses. 50%, 75%, and 95% tolerance ellipses generated of the entire group of synchronized swimmers. R/h, height-adjusted resistance; Xc/h, height-adjusted reactance.

Fig 4 shows the 50%, 75% and 95% tolerance ellipses corresponding to the whole SS sample (C_o_ and J_r_ together): R/h = 319.7 ± 36.7 Ω/m; Xc/h = 39.9 ± 3.9 Ω/m; *r* = 0.78.

### Pre-post differences

The BIA vector migration (Fig 2) was characterized by an increase in R/h and Xc/h, indicating mild dehydration after training both in C_o_ (*T*^2^ = 82.1) and J_r_ (*T*^*2*^ = 41.8) (*p* < 0.001). This was paralleled by a decrease in BM in both groups of swimmers (*p* = 0.0001) as shown in Table 3. In contrast, all bioelectrical variables significantly increased (Table 3).

**Table 3:** Anthropometric and bioelectrical parameters before (Pre) and after (Post) training.

		Pre _(95% CI)_	Post _(95% CI)_	Δ-value	Paired t-test	*p*-value	Cohen’s d
				%	*t*	*p*	*d*
**Comen (n = 34)**		** **	** **	** **	** **	** **	** **
Anthropometric							** **
	BM (kg)	47.2 ± 7.0 _(44.8–49.7)_	46.9 ± 7.0 _(44.5–49.3)_	-0.8 ± 0.6	8.081	0.0001^*^	0.20^a^
Bioelectrical							** **
	R (Ω)	529.5 ± 46.1 _(513.4–545.6)_	548.8 ± 48.5 _(531.9–565.7)_	3.7 ± 3.0	-7.251	0.0001^*^	0.39^b^
	Xc (Ω)	64.4 ± 5.1 _(62.7–66.2)_	67.7 ± 5.0 _(66.0–69.5)_	5.2 ± 3.3	-9.193	0.0001^*^	0.67^c^
	R/h (Ω/m)	328.4 ± 38.8 _(314.9–341.9)_	340.5 ± 41.0 _(326.1–354.8)_	3.7 ± 3.0	-7.104	0.0001^*^	0.30^b^
	Xc/h (Ω/m)	40.0 ± 4.5 _(38.4–41.5)_	42.0 ± 4.6 _(40.4–43.6)_	5.2 ± 3.3	-8.905	0.0001^*^	0.43^b^
	PA (Ω)	7.0 ± 0.5 _(6.8–7.1)_	7.1 ± 0.5 _(6.9–7.2)_	1.5 ± 2.5	-2.863	0.007^*^	0.20^a^
	Z (Ω/m)	330.9 ± 38.9 _(317.2–344.4)_	343.1 ± 41.2 _(328.7–357.4)_	3.7 ± 3.0	-7.178	0.0001^*^	0.20^a^
	*r* (R/h, Xc/h)	0.84	0.84	—	—	—	
**Junior (n = 15)**		** **	** **	** **	** **	** **	** **
Anthropometric							** **
	BM (kg)	53.5 ± 5.2 _(50.6–56.3)_	53.2 ± 5.1 _(50.3–56.0)_	-0.6 ± 0.4	4.634	0.0001^*^	0.17^a^
Bioelectrical							** **
	R (Ω)	498.5 ± 35.1 _(479.1–518.0)_	518.5 ± 38.9 _(497.0–540.1)_	4.0 ± 3.3	-4.870	0.0001^*^	0.53^c^
	Xc (Ω)	65.8 ± 2.9 _(64.2–67.4)_	70.2 ± 4.8 _(67.5–72.8)_	6.6 ± 3.9	-6.447	0.0001^*^	0.82^c^
	R/h (Ω/m)	299.9 ± 21.6 _(287.9–311.9)_	311.9 ± 23.4 _(298.9–324.9)_	4.0 ± 3.3	-4.864	0.0001^*^	0.53^c^
	Xc/h (Ω/m)	39.6 ± 2.2 _(38.4–40.8)_	42.2 ± 3.4 _(40.3–44.1)_	6.6 ± 3.9	-6.352	0.0001^*^	0.62^c^
	PA (Ω)	7.5 ± 0.4 _(7.3–7.8)_	7.7 ± 0.4 _(7.5–7.9)_	2.4 ± 3.3	-2.909	0.011^*^	0.45^b^
	Z (Ω/m)	302.5 ± 21.7 _(290.5–314.5)_	314.8 ± 23.5 _(301.7–327.8)_	4.1 ± 3.3	-4.928	0.0001^*^	0.51^c^
	r (R/h, Xc/h)	0.66	0.76	—	—	—	

Values are the mean ± standard deviation; BM, body mass; R, resistance; Xc, reactance; h, height; PA, phase angle; Z, impedance vector module; r, Pearson correlation coefficient between R/h and Xc/h; %Δ, percent differences Pre to Post; CI, 95% confidence interval

*significant differences between Pre and Post, p-value < 0.05 (paired t-test Pre vs. Post); a, small effect size (≤ 0.2); b, medium effect size (d ≤ 0.5); c, large effect size (d ≤ 0.8).

### BIVA correlations

A positive correlation (*r* = 0.45, *p* = 0.001) was found between PA and chronological age in the whole SS sample. Additionally, PA was negatively related (*r* = -0.91; *p* < 0.001) to the ECW:TBW. No correlations were observed between bioelectrical pre to post changes in relation to BM.

## Discussion

This study showed that synchronized swimmers experienced a modest level of dehydration after an intense training session (BM loss ~0.6–0.8% BM) that was detected by BIVA. In addition, we report a specific BIA vector distribution in these young elite SS swimmers in comparison with a healthy, nonathletic reference population of similar age. In fact, this is the first time that specific reference distribution ellipses in a female sporting group is being reported (Fig 4).

BIVA allows for an analysis of both the homeostatic state and possible BIA vector migration, arising from any variation in body fluid [12, 26]. Nowadays, BIVA is a widely used technique in medicine as a valid tool in the assessment of different physiological states and clinical conditions in which euhydration is frequently altered, such as renal disease [41], critically ill patients [42], pulmonary disease [43], heart failure [44], gastrointestinal disease [45], and pregnancy and postpartum [46]. Its properties are especially interesting for hydration assessments in both the training process and competitive sporting events [16, 47].

Nevertheless, in protocols measuring parameters before and after exercise to analyze acute vector shifts, certain factors that may generate errors in the bioelectrical signal should be controlled in order to provide accurate and reliable results, including: skin preparation [48]; previous hydration status [49]; previous consumption of food or beverage [35, 50, 51]; body position and posture during measurements [21, 51, 52]; electrode impedance [53], position and placement modification [51, 52]; time of body fluid stabilization [54, 55]; variations in cutaneous blood flow and temperature [35, 52]; skin electrolyte accumulation produced by physical exercise [35]; reproducibility of bioelectrical measurements influenced by biological intra-day [56] and inter-day variations [50]; environmental conditions [21, 52]; menstrual cycle [17, 57]; and injury condition [58].

Despite the ecological design of this research, the study protocol attempted to control these factors. As mentioned above, ingestion of a meal or beverage has an influence on Z, which may decrease over a 2-to 4-h period after a meal, generally representing a change of < 3% in Z values [22]. Therefore, in our study, post-exercise BIVA measurements could have been influenced by breakfast and water intake in the middle of the training session, possibly underestimating Z values by ~9–10 Ω. The amount of water intake at the end of the training should not have affected the BIVA measurements because the recent ingestion of a meal or beverage (< 1 h from the ingestion to BIA measurements) appears to be "electrically silent" and to have a minimal effect on whole- body Z [59]. With regard to the temperature control, it is known that every 1.0°C increase in the skin can lead to a decrease in R of up to ~11% [60]. Possible pre-post BIA differences related to environmental and cutaneous temperatures of the swimmers were controlled, accepting increases or decreases lower than 1°C as, in this range of values, differences in Z appear not to be significant [61]. Finally, it should be noted that the study sample was composed of female athletes. Thus, to minimize the body fluid fluctuations caused by the effect of female hormonal kinetics [17] and consequent changes in performance ability [62], international recommendations were followed [28, 57]. Thus, those swimmers who were in a premenstrual phase (luteal or secretory phase) or who were taking contraceptives and/or menstrual cycle pharmacologic regulators were excluded.

### BIA vector changes evoked by training

This study is the first to use BIVA to characterize variations in hydration status in young SS athletes evoked by training. RXc paired graphs showed significant vector changes after exercise in both groups (Fig 2), which were interpreted as mild dehydration (average loss <1% BM) [63]. Nevertheless, no correlation was observed between changes in BM and BIA vector migration. This could be due to the fluid intake of the athletes during training, which was maintained due to the ecological study design. A recent investigation in which no food/fluid intake was allowed found similar results with exercise-induced dehydration [29]. The researchers noted that this could be influenced by inadequate criteria for stable bioelectrical impedance measurements or by exercise-related factors, such as sweat rate, respiratory water loss and oxidative water production, that may lead to BM loss without an effective net negative fluid balance [64]. However, these results differ from other studies [65, 66] that found a significant relationship between changes in bioelectrical values and BM induced passively and/or chronically. It is possible that greater changes could have been observed if the swimmers had not ingested fluids during the long and intense training (Table 2). Nevertheless, no significant relationships were found with BM in the present study.

Only two studies in the literature have investigated short-term vector changes induced by exercise. Collodel et al. [67] did not find differences in R and Xc—and thus in vector position—after an incremental maximal cycle ergometer test (of an unspecified duration) performed by healthy sedentary subjects and moderately trained subjects, although both the BM and the hematocrit experienced significant changes. Nevertheless, two possible limitations could have influenced their bioelectrical results: the post-exercise measurement was performed 5 min after finishing the test; thus, some previously mentioned sources of error may have influenced the bioelectrical signal [35]. Furthermore, an RXc mean graph was reported for pre-post analysis, instead of an RXc paired graph. The type of Hotelling’s T^2^ test chosen should have also been clarified because the RXc paired graph and the paired one-sample Hotelling’s T^2^ test are the appropriate analyses in this case, and they may have given different results. Conversely, Gatterer et al. [29] analyzed the short-term bioelectrical changes in well-trained subjects after 1 h of a self-rated intensity cycle ergometer test in the heat (environmental chamber). The authors reported findings similar to those of the present study, observing increased resistance and reactance, as well as a vector migration, after exercise in the heat.

In our study, vector migration along the major axis due to increased R/h and Xc/h indicates fluid loss (Fig 2), as the length of the vector is inversely related to TBW [13]. Resistance is pure opposition of the conductor to the flow of current [52]. Therefore, the significantly increased resistance experienced by the swimmers reflects the decreased body fluids [35], which is supported by the decrease in BM, and is also probably followed—which we cannot prove—by changes in electrolyte concentration [66]. With regard to the reactance, Gatterer et al. [29] suggested that the increased reactance after exercise could indicate fluid shifts between intra- and extracellular compartments. Xc maintains a relationship with cell membrane capacitance (Cm), which is affected by the size, thickness and composition of the cell membranes [68]. Alterations such as fluid shifts between compartments induced by physical activity modify the characteristics of the muscle cells. As suggested, the cell membrane becomes thinner as the cell swells and Cm increases, and the opposite occurs when the cell shrinks [69], thus affecting Xc. Furthermore, as proposed by De Lorenzo et al. [70], variations in fluid distribution would modify the characteristic frequency (Fc)—i.e., the frequency at which Xc is maximal). Because Xc is highly dependent on the relationship between the frequency of measurement and Fc, changes in Fc evoke great variations in Xc at 50 kHz, simply because this frequency is a fixed point on the changing impedance locus [71]. However, De Lorenzo and colleagues’ hypothesis should be considered with caution because it refers to Hanai’s model, which relays on assumptions such as spherical cells shape. Nevertheless, the meaning of Xc behavior after exercise remains to be clarified. In this regard, consideration of the Xc as an indicator of dielectric mass (membranes and tissue interfaces) of soft tissues [52] should be reviewed, as it may not be applicable in this type of protocols. It should be noted that despite the fact that vector changes after fluid removal and overload (e.g., the wet–dry cycle of dialysis) as a non-physiological process is clinically well-established [13], every dehydration process induced by physical exercise is a consequence of several physiological adaptations whose relationship with the vector behavior is scarcely explored, especially at the cellular level and considering the kinetics of Xc.

Because BIVA appears to be sensitive to body water adaptations evoked by high intensity SS training, it could help to assess hydration variations in real time and could also substitute the current hydration biomarkers that require a mobile laboratory. Nevertheless, we are still far from confirming BIVA as a valid and reliable biomarker of hydration status. Its progressive use as a complementary measure to hematological hydration indicators will allow us to parameterize its values and demonstrate its real possibilities in the near future.

### Determinants of BIA vector distribution pattern in synchronized swimmers

The individual anthropometric dimensions, such as weight and height, determine the body’s bioelectrical properties [52]. Although bioelectrical variables are normalized for height, BMI calculation enables better contextualization of the sample. The results of the BMI in both groups (Table 1)—underweight and normal weight in C_o_ and J_r_ swimmers, respectively—seem to be coherent with the great physiological demands of this sport [4]. These anthropometric characteristics are necessary to understand that the bioelectrical signal will also be specifically related to each sport, sex and age [15]. The comparison of BIA vector distribution values of SS athletes with that of other sports practitioners is difficult due to the absence of values for female athletes and differences in age and gender with regard to the only study that, to our knowledge, has provided a characterization of a sport-specific population, i.e. male soccer players [47]. Nevertheless, a comparison between SS and reference populations or between both groups of swimmers according to their age can be discussed.

#### Characterization of synchronized swimmers

In the present group of swimmers, PA variation was positively correlated with age, following a trend similar to that of the general athletic population of the same sex and age, in accordance with Koury et al. [15]. This positive correlation in athletes is in agreement with the increase in metabolic tissues during biological maturation [39]. Mean and individual Z vectors (Fig 2) were found to be displaced to the left and mostly scattered outside the 75% tolerance ellipse (in many cases, outside the 95% tolerance ellipse) on the RXc graphs when swimmers were compared to the reference nonathletic Italian female population of similar age [39]. Furthermore, with increasing age and performance level of the athletes (Fig 3), a displacement to the left was also observed, due to a decrease in the R/h component in the absence of a difference in the Xc/h component. Other studies [15, 29, 47] have also reported vectors of sport samples shifted to the left when compared to their reference populations, which might reflect the specific adaptations of body composition in different sports [72]. Additionally, vectors shifted to the left have been reported with increasing age [15] and performance level [47] in sport samples. It remains to be investigated whether the differences are the result of vector displacement due to biological maturation, to the specific training process or a combination of both.

Athletes generally possess increased soft tissue mass and differing fluid content compared to the sedentary population [72]. Total body fluid is affected by factors such as training [73]. Trained athletes have a greater amount of body fluid and different fluid distribution between the intracellular and extracellular compartments. This can be because of their larger muscle mass, increased plasma volume and muscle glycogen reserves [8, 35], which could increase water transport into the muscle [74] and fluid-regulating hormone adaptations (i.e., aldosterone and sensitivity) [75].

As suggested, both the increased BCM in SS indicated by the BIA vector and the vector differences due to decreased R/h with similar Xc/h values could reflect different intracellular water (ICW) content. On a related note, and according to Chertow et al. [31], a negative relationship was found between the ECW:TBW and PA in the present study. Because SS showed a greater PA, the greater ICW content of the swimmers compared to the reference population—as well as J_r_ compared to C_o_—is likely due to the hypertrophy of the muscle fibers [47]. Additionally, the greater PA could also reflect better cell function [12].

Thus, the present findings highlight the need for specific new tolerance ellipses for the SS sporting population (Fig 4). These ellipses might be useful for interpretation of individual vectors and for defining target regions of impedance vectors for lower-level SS athletes. Nevertheless, further studies should increase the sample size and analyze different performance levels; this will help determine whether specific training activity may induce vector migration to the side in the higher level swimmers, as well as the utility of the tolerance ellipses for monitoring hydration status and performance state.

The main limitation of the present study, in addition to those previously mentioned for the sake of text fluency, is the previously mentioned ecological constraints of the protocol, which may have caused an attenuation of the bioelectrical changes after training. Additionally, with regard to the comparison of the SS sample to the reference population, no tolerance ellipses of the healthy reference population have been published for this specific age range. Thus, this study used the tolerance ellipses of the healthy reference population closest in age.

In conclusion, BIVA appears to be sensitive to hydration changes evoked by high intensity SS training, regardless of age and performance level. Moreover, the present study showed that SS swimmers are characterized by a specific distribution of BIVA parameters when compared to a healthy nonathletic reference population. Furthermore, BIVA also showed differences between swimmers of different age and performance level. This is the first time that specific tolerance ellipses in a female sport group are being reported.

The use of BIVA as an indicator of dehydration in sport practice is clearly an emerging research area. Beyond the need for further validation of this methodology, especially in pre- to post-exercise designs, generation of new ellipses according to each sport, age, sex, race and sport level is needed in order to establish useful and comparable reference values for the field of sport sciences.

## Supporting information

S1 Dataset Study database. (XLSX)

## Abbreviations

BCM: Body cell mass
BIA: Bioelectrical impedance analysis
BMI: Body mass index
BIVA: Bioelectrical impedance vector analysis
ECW: Extracellular water
ECW:TBW: Extracellular water/total body water ratio
Fc: Characteristic frequency
FFM: Fat-free mass
FM: Fat mass
h: Body height
Hotelling’s T^2^: Test comparing mean group vectors
ICW: Intracellular water
Cm: Membrane capacitance
PA: Phase angle
R: Bioelectrical resistance (R/h if adjusted by height)
RXc graph: R/h vs. Xc/h probabilistic plot
SD: Standard deviation
SF-BIA: Single-frequency bioelectrical impedance analysis
s-RPE: Session rating of pereceived exertion
SS: Synchronized swimmming
TBW: Total body water
Xc: Bioelectrical reactance (Xc/h if adjusted by height)
Z: Bioelectrical impedance
Z vector: Vector yield by the RXc graph
°C_core_, °C_hand,_ °C_foot_: Core and skin temperatures of the right hand and foot

## References

[1] Jamnik V. An evaluation of the physiological response to competitive synchronized swimming and the physiological characteristics of elite synchronized swimmers: Toronto, ON: York University; 1987.

[2] Mountjoy M. Injuries and medical issues in synchronized Olympic sports. Curr Sports Med Rep. 2009;8(5):255–61. doi: 10.1249/JSR.0b013e3181b84a09 19741353 10.1249/JSR.0b013e3181b84a09

[3] Mountjoy M. The basics of synchronized swimming and its injuries. Clin Sports Med. 1999;18(2):321–36. 10230568 10.1016/s0278-5919(05)70148-4

[4] Rodríguez-Zamora L, Iglesias X, Barrero A, Chaverri D, Erola P, Rodríguez FA. Physiological responses in relation to performance during competition in elite synchronized swimmers. PLoS One. 2012;7(11):e49098 doi: 10.1371/journal.pone.0049098 23155452 10.1371/journal.pone.0049098 PMC3498322

[5] Bar-Or O, Dotan R, Inbar O, Rotshtein A, Zonder H. Voluntary hypohydration in 10-to 12-year-old boys. J Appl Physiol (1985). 1980;48(1):104–8. 10.1152/jappl.1980.48.1.104 7353962

[6] Cheuvront SN, Kenefick RW, Montain SJ, Sawka MN. Mechanisms of aerobic performance impairment with heat stress and dehydration. J Appl Physiol (1985). 2010;109(6):1989–95. 20689090 10.1152/japplphysiol.00367.2010

[7] Montain SJ, Coyle EF. Influence of graded dehydration on hyperthermia and cardiovascular drift during exercise. J Appl Physiol (1985). 1992;73(4):1340–50. 1447078 10.1152/jappl.1992.73.4.1340

[8] Meyer F, Volterman KA, Timmons BW, Wilk B. Fluid balance and dehydration in the young athlete assessment considerations and effects on health and performance. Am J Lifestyle Med. 2012;6(6):489–501.

[9] Schaal K, Tiollier E, Le Meur Y, Casazza G, Hausswirth C. Elite synchronized swimmers display decreased energy availability during intensified training. Scand J Med Sci Sports. 2016 doi: 10.1111/sms.12716 10.1111/sms.12716 27367601

[10] Lundy B. Nutrition for synchronized swimming: a review. Int J Sport Nutr Exerc Metab. 2011;21:436–45. 21904005 10.1123/ijsnem.21.5.436

[11] McGarvey J, Thompson J, Hanna C, Noakes TD, Stewart J, Speedy D. Sensitivity and specificity of clinical signs for assessment of dehydration in endurance athletes. Br J Sports Med. 2010;44(10):716–9. doi: 10.1136/bjsm.2008.053249 18981042 10.1136/bjsm.2008.053249

[12] Norman K, Stobäus N, Pirlich M, Bosy-Westphal A. Bioelectrical phase angle and impedance vector analysis—Clinical relevance and applicability of impedance parameters. Clin Nutr. 2012;31(6):854–61. doi: 10.1016/j.clnu.2012.05.008 22698802 10.1016/j.clnu.2012.05.008

[13] Lukaski HC, Piccoli A. Bioelectrical impedance vector analysis for assessment of hydration in physiological states and clinical conditions In: Preedy V, editor. Handbook of Anthropometry. London: Springer; 2012 p. 287–305.

[14] Piccoli A, Rossi B, Pillon L, Bucciante G. A new method for monitoring body fluid variation by bioimpedance analysis: the RXc graph. Kidney Int. 1994;46(2):534–9. 7967368 10.1038/ki.1994.305

[15] Koury J, Trugo N, Torres A. Phase angle and bioelectrical impedance vectors in adolescent and adult male athletes. Int J Sports Physiol Perform. 2014;9(5):798–804. doi: 10.1123/ijspp.2013-0397 24414089 10.1123/ijspp.2013-0397

[16] Mascherini G, Gatterer H, Lukaski H, Burtscher M, Galanti G. Changes in hydration, body-cell mass and endurance performance of professional soccer players through a competitive season. J Sports Med Phys Fitness. 2015;55(7–8):749–55. 25303072

[17] Gleichauf C, Roe D. The menstrual cycle's effect on the reliability of bioimpedance measurements for assessing body composition. Am J Clin Nutr. 1989;50(5):903–7. 2816797 10.1093/ajcn/50.5.903

[18] WMA. World Medical Association Declaration of Helsinki: ethical principles for medical research involving human subjects. JAMA. 2013;310(20):2191–4. doi: 10.1001/jama.2013.281053 24141714 10.1001/jama.2013.281053

[19] Fortes MB, Diment BC, Di Felice U, Walsh NP. Dehydration decreases saliva antimicrobial proteins important for mucosal immunity. Appl Physiol Nutr Metab. 2012;37(5):850–9. doi: 10.1139/h2012-054 22686429 10.1139/h2012-054

[20] Aranceta J, Serra-Majem L, Ribas L, Perez-Rodrigo C. Breakfast consumption in Spanish children and young people. Public Health Nutr. 2001;4(6a):1439–44. 11918497 10.1079/phn2001235

[21] Rush E, Crowley J, Freitas I, Luke A. Validity of hand-to-foot measurement of bioimpedance: standing compared with lying position. Obesity. 2006;14(2):252–7. doi: 10.1038/oby.2006.32 16571850 10.1038/oby.2006.32

[22] Kushner R, Gudivaka R, Schoeller D. Clinical characteristics influencing bioelectrical impedance analysis measurements. Am J Clin Nutr. 1996;64(3):423S–7S. 8780358 10.1093/ajcn/64.3.423S

[23] Lukaski HC, Bolonchuk WW, Siders WA, Hall CB. Body composition assessment of athletes using bioelectrical impedance measurements. J Sports Med Phys Fitness. 1990;30(4):434–40. 2079851

[24] Borg G, Hassmén P, Lagerström M. Perceived exertion related to heart rate and blood lactate during arm and leg exercise. Eur J Appl Physiol Occup Physiol. 1987;56(6):679–85. 3678222 10.1007/BF00424810

[25] Stewart A, Marfell-Jones M, Olds T, de Ridder H. International standards for anthropometric assessment. Murcia, Spain: International Society for the Advancement of Kinanthropometry 2011.

[26] Piccoli A. Bioelectric impedance measurement for fluid status assessment. Contrib Nephrol. 2010;164:143–52. doi: 10.1159/000313727 20428000 10.1159/000313727

[27] Moreno MV, Ribbe-Cornet E, Rebeyrol J, Vannicatte A, Krief L. Evaluation of a new impedancemeter to independently measure extracellular, intracellular and total body water volumes: application to the measurement of hydration. Med Biol Eng Comput. 2015;53(10):989–99. doi: 10.1007/s11517-015-1305-8 26036775 10.1007/s11517-015-1305-8

[28] Yanovski SZ, Hubbard VS, Heymsfield SB, Lukaski HC. Bioelectrical impedance analysis in body composition measurement: National Institutes of Health technology assessment conference statement. Am J Clin Nutr. 1996;64(3):524S–32S. 8780375 10.1093/ajcn/64.3.524S

[29] Gatterer H, Schenk K, Laninschegg L, Schlemmer P, Lukaski H, Burtscher M. Bioimpedance identifies body fluid loss after exercise in the heat: a pilot study with body cooling. PLoS One. 2014;9(10):e109729 doi: 10.1371/journal.pone.0109729 25279660 10.1371/journal.pone.0109729 PMC4184898

[30] Goovaerts H, Faes TJ, De Valk-De Roo G, Ten Bolscher M, Netelenbosch J, Van der Vijgh W, et al Extra-cellular volume estimation by electrical impedance-phase measurement or curve fitting: a comparative study. Physiol Meas. 1998;19(4):517 9863677 10.1088/0967-3334/19/4/006

[31] Chertow GM, Lowrie EG, Wilmore DW, Gonzalez J, Lew NL, Ling J, et al Nutritional assessment with bioelectrical impedance analysis in maintenance hemodialysis patients. J Am Soc Nephrol. 1995;6(1):75–81. 7579073 10.1681/ASN.V6175

[32] Piccoli A. Whole body-single frequency bioimpedance. Contrib Nephrol. 2005;149:150–61. doi: 10.1159/000085478 15876839 10.1159/000085478

[33] Pillon L, Piccoli A, inventors. RXc graph and RXc Z-score graph methods. United States patent application US 10/740,911. 2003 Dec 18.

[34] Peiffer JJ, Abbiss CR, Nosaka K, Peake JM, Laursen PB. Effect of cold water immersion after exercise in the heat on muscle function, body temperatures, and vessel diameter. J Sci Med Sport. 2009;12(1):91–6. doi: 10.1016/j.jsams.2007.10.011 18083634 10.1016/j.jsams.2007.10.011

[35] O'Brien C, Young A, Sawka M. Bioelectrical impedance to estimate changes in hydration status. Int J Sports Med. 2002;23(5):361–6. doi: 10.1055/s-2002-33145 12165888 10.1055/s-2002-33145

[36] Foster C, Daines E, Hector L, Snyder AC, Welsh R. Athletic performance in relation to training load. Wis Med J. 1996;95(6):370–4. 8693756

[37] Psycharakis SG. A longitudinal analysis on the validity and reliability of ratings of perceived exertion for elite swimmers. J Strength Cond Res. 2011;25(2):420–6. doi: 10.1519/JSC.0b013e3181bff58c 20351574 10.1519/JSC.0b013e3181bff58c

[38] Piccoli A, Pastori G. BIVA software. Padova: Department of Medical and Surgical Sciences, University of Padova, Italy 2002.

[39] De Palo T, Messina G, Edefonti A, Perfumo F, Pisanello L, Peruzzi L, et al Normal values of the bioelectrical impedance vector in childhood and puberty. Nutrition. 2000;16(6):417–24. 10869896 10.1016/s0899-9007(00)00269-0

[40] Cohen J. A power primer. Psychol Bull. 1992;112(1):155–9. 19565683 10.1037//0033-2909.112.1.155

[41] Piccoli A. Bioelectric impedance vector distribution in peritoneal dialysis patients with different hydration status. Kidney Int. 2004;65(3):1050–63. doi: 10.1111/j.1523-1755.2004.00467.x 14871426 10.1111/j.1523-1755.2004.00467.x

[42] Baldwin CE, Paratz JD, Bersten AD. Body composition analysis in critically ill survivors: a comparison of bioelectrical impedance spectroscopy devices. JPEN J Parenter Enteral Nutr. 2012;36(3):306–15. doi: 10.1177/0148607111433055 22318964 10.1177/0148607111433055

[43] Walter-Kroker A, Kroker A, Mattiucci-Guehlke M, Glaab T. A practical guide to bioelectrical impedance analysis using the example of chronic obstructive pulmonary disease. Nutr J. 2011;10(1):1. 21510854 10.1186/1475-2891-10-35 PMC3110108

[44] Castillo-Martinez L, Colin-Ramirez E, Orea-Tejeda A, Gonzalez Islas DG, Rodriguez Garcia WD, Santillan Diaz C, et al Cachexia assessed by bioimpedance vector analysis as a prognostic indicator in chronic stable heart failure patients. Nutrition. 2012;28(9):886–91. doi: 10.1016/j.nut.2011.11.024 22480798 10.1016/j.nut.2011.11.024

[45] Norman K, Smoliner C, Kilbert A, Valentini L, Lochs H, Pirlich M. Disease-related malnutrition but not underweight by BMI is reflected by disturbed electric tissue properties in the bioelectrical impedance vector analysis. Br J Nutr. 2008;100(3):590–5. doi: 10.1017/S0007114508911545 18234142 10.1017/S0007114508911545

[46] Lukaski HC, Hall CB, Siders WA. Assessment of change in hydration in women during pregnancy and postpartum with bioelectrical impedance vectors. Nutrition. 2007;23(7–8):543–50. doi: 10.1016/j.nut.2007.05.001 17570642 10.1016/j.nut.2007.05.001

[47] Micheli ML, Pagani L, Marella M, Gulisano M, Piccoli A, Angelini F, et al Bioimpedance and Impedance Vector Patterns as Predictors of League Level in Male Soccer Players. Int J Sports Physiol Perform. 2014;9(3):532–9. doi: 10.1123/ijspp.2013-0119 23881291 10.1123/ijspp.2013-0119

[48] Laferriere P, Lemaire ED, Chan AD. Surface electromyographic signals using dry electrodes. IEEE Trans Instrum Meas. 2011;60(10):3259–68.

[49] Berneis K, Keller U. Bioelectrical impedance analysis during acute changes of extracellular osmolality in man. Clin Nutr. 2000;19(5):361–6. doi: 10.1054/clnu.2000.0133 11031076 10.1054/clnu.2000.0133

[50] Deurenberg P, Weststrate JA, Paymans I, Van der Kooy K. Factors affecting bioelectrical impedance measurements in humans. Eur J Clin Nutr. 1988;42(12):1017–22. 3234328

[51] Gualdi-Russo E, Toselli S. Influence of various factors on the measurement of multifrequency bioimpedance. Homo. 2002;53(1):1–16. 12365353 10.1078/0018-442x-00035

[52] Lukaski HC. Biological indexes considered in the derivation of the bioelectrical impedance analysis. Am J Clin Nutr. 1996;64(3):397S–404S. 8780355 10.1093/ajcn/64.3.397S

[53] Nescolarde L, Lukaski H, De Lorenzo A, de-Mateo-Silleras B, Redondo-Del-Rio MP, Camina-Martin MA. Different displacement of bioimpedance vector due to Ag/AgCl electrode effect. Eur J Clin Nutr. 2016;70(12):1401–7. doi: 10.1038/ejcn.2016.121 27380885 10.1038/ejcn.2016.121

[54] Slinde F, Bark A, Jansson J, Rossander-Hulthén L. Bioelectrical impedance variation in healthy subjects during 12 h in the supine position. Clin Nutr. 2003;22(2):153–7. 12706132 10.1054/clnu.2002.0616

[55] Zhu F, Schneditz D, Wang E, Levin NW. Dynamics of segmental extracellular volumes during changes in body position by bioimpedance analysis. J Appl Physiol (1985). 1998;85(2):497–504. 9688726 10.1152/jappl.1998.85.2.497

[56] Rodríguez G, Moreno LA, Sarría A, Fleta J, Bueno M. Assessment of nutritional status and body composition in children using physical anthropometry and bioelectrical impedance: influence of diurnal variations. J Pediatr Gastroenterol Nutr. 2000;30(3):305–9. 10749416 10.1097/00005176-200003000-00017

[57] Lusseveld E, Peters ETJ, Deurenberg P. Multifrequency bioelectrical impedance as a measure of differences in body water distribution. Ann Nutr Metab. 1993;37(1):44–51. 8470872 10.1159/000177748

[58] Nescolarde L, Yanguas J, Lukaski H, Alomar X, Rosell-Ferrer J, Rodas G. Effects of muscle injury severity on localized bioimpedance measurements. Physiol Meas. 2015;36(1):27–42. doi: 10.1088/0967-3334/36/1/27 25500910 10.1088/0967-3334/36/1/27

[59] Evans W, McClagish H, Trudgett C. Factors affecting the in vivo precision of bioelectrical impedance analysis. Appl Radiat Isot. 1998;49(5):485–7. 9569522 10.1016/s0969-8043(97)00061-4

[60] Caton J, Mole P, Adams W, Heustis D. Body composition analysis by bioelectrical impedance: effect of skin temperature. Med Sci Sports Exerc. 1988;20(5):489–91. 3193865

[61] Liang M, Norris S. Effects of skin blood flow and temperature on bioelectric impedance after exercise. Med Sci Sports Exerc. 1993;25(11):1231–9. 8289609

[62] McKee JE, Cameron N. Bioelectrical impedance changes during the menstrual cycle. Am J Hum Biol. 1997;9(2):155–61. 28561518 10.1002/(SICI)1520-6300(1997)9:2<155::AID-AJHB1>3.0.CO;2-#

[63] Armstrong L, Rosenberg I, Armstrong L, Manz F, Dal Canton A, Barclay D, et al Hydration assessment techniques. Nutr Rev. 2005;63(6 II):S40–S54. doi: 10.1301/nr.2005.jun.S40-S54 16028571 10.1111/j.1753-4887.2005.tb00153.x

[64] Maughan RJ, Shirreffs SM, Leiper JB. Errors in the estimation of hydration status from changes in body mass. J Sports Sci. 2007;25(7):797–804. doi: 10.1080/02640410600875143 17454547 10.1080/02640410600875143

[65] Gatterer H, Wille M, Faulhaber M, Lukaski H, Melmer A, Ebenbichler C, et al Association between body water status and acute mountain sickness. PLoS One. 2013;8(8):e73185 doi: 10.1371/journal.pone.0073185 24013267 10.1371/journal.pone.0073185 PMC3754926

[66] Piccoli A, Piazza P, Noventa D, Pillon L, Zaccaria M. A new method for monitoring hydration at high altitude by bioimpedance analysis. Med Sci Sports Exerc. 1996;28(12):1517–22. 8970147 10.1097/00005768-199612000-00012

[67] Collodel L, Favretto G, Teodori T, Caenaro G, Mordacchini M, Stritoni P, et al Use of bioelectrical impedance analysis for monitoring fluid shift during maximal aerobic exercise. Med Sport. 1997;50(2):197–202.

[68] Sperelakis N. Origin of resting membrane potentials In: Sperelakis N, editor. Cell physiology source book: essentials of membrane biophysics. London: Academic Press; 2012 p. 123.

[69] Gerth WA, Montgomery LD, Wu Y-C, editors. A computer-based bioelectrical impedance spectroscopic system for noninvasive assessment of compartmental fluid redistribution. Third IEEE Symposium on Computer-Based Medical Systems. 1990:446–453.

[70] De Lorenzo A, Andreoli A, Matthie J, Withers P. Predicting body cell mass with bioimpedance by using theoretical methods: a technological review. J Appl Physiol (1985). 1997;82(5):1542–58. 9134904 10.1152/jappl.1997.82.5.1542

[71] Lofgren B. The electrical impedance of a complex tissue and its relation to changes in volume and fluid distribution; a study on rat kidneys. Acta Physiol Scand Suppl. 1951;81:1–51. 14837765

[72] Andreoli A, Monteleone M, Van Loan M, Promenzio L, Tarantino U, De Lorenzo A. Effects of different sports on bone density and muscle mass in highly trained athletes. Med Sci Sports Exerc. 2001;33(4):507–11. 11283423 10.1097/00005768-200104000-00001

[73] Convertino VA. Blood volume: its adaptation to endurance training. Med Sci Sports Exerc. 1991;23(12):1338–48. 1798375

[74] Sawka MN. Physiological consequences of hypohydration: exercise performance and thermoregulation. Med Sci Sports Exerc. 1992;24(6):657–70. 1602938

[75] Fellmann N. Hormonal and plasma volume alterations following endurance exercise. Sports Med. 1992;13(1):37–49. 1553454 10.2165/00007256-199213010-00004

